# Structure stability of (U, Pu) C and (U, Pu) N compositions

**DOI:** 10.1038/s41598-025-03910-y

**Published:** 2025-06-06

**Authors:** William A. Watson, Sophie Cooper, Matthew Horton, Robin W. Grimes

**Affiliations:** 1 Department of Materials, Imperial College London, London, SW7 2AZ UK; 2 United Kingdom National Nuclear Laboratory Limited, Chadwick House, Birchwood Park, Warrington, WA3 6AE UK

**Keywords:** UN, PuN, UC, PuC, DFT, Structure stability, Atomistic models, Nuclear fuel

## Abstract

Atomic scale computer simulations based on density functional theory (DFT) are used to calculate the formation energies and structures associated with phases in the U–N, Pu–N, U–C and Pu–C systems. Stable phases across the compositional spaces, from the metal to the nitrogen gas or graphite end members, are identified using convex hull analysis. Many predicted phases correspond to those known from experimental phase diagrams (e.g. UN, U_2_N_3_; PuN; UC, U_2_C_3_; Pu_2_C_3_). However, many phases only sit on the convex hull upon inclusion of a suitably characterised Hubbard parameter (i.e. DFT + U). A nonstoichiometric composition of UN_2−x_ is identified on the U–N convex hull but others, including stoichiometric UN_2_, are close to the line. A stoichiometric structure for Pu_3_C_2_ with $R\overline{3}c$ symmetry is identified, alongside which a nonstoichiometric PuC_1−x_ phase has a similar energy.

## Introduction

Uranium and plutonium carbide (U/PuC) and nitride (U/PuN) nuclear fuels have been developed since the 1960s, being proposed as alternatives to oxides, especially for fast neutron spectrum reactors^1–3^. More recently, they have been considered for advanced Generation IV reactors^4,5^, such as high-temperature gas-cooled reactors (HTGRs), and have been utilised in the production of TRISO fuel particles^6,7^. This is motivated by the fact, in comparison to conventional oxide fuels, nitrides and carbides exhibit higher thermal conductivity and higher fissile element density while still exhibiting high melting points and similar radiation tolerance^4,8^. The beneficial properties of UN, PuN, UC and PuC, compared to conventional oxide fuels, could allow the fuel to operate at higher burn-ups. UN and UC have already undergone irradiation testing in both conventional fuel pin and TRISO particle forms^9,10^, with the latter compromising UC in a uranium oxycarbide (UCO) fuel kernel mixture. PuN and PuC irradiation testing is significantly less mature with experimental studies largely focusing on uranium–plutonium nitride and carbide fuel mixtures^11–13^. The inclusion of these Pu structures supports further assessment of the UK plutonium stockpile as a fuel asset for Generation IV reactors.

While most studies have focused on the mononitrides and monocarbides, it may be possible to use other compositions for fuel applications. It is also important to understand the stability of possible phases to support fuel synthesis in achieving a particular desired phase. Also, if the operational envelope extends to higher temperatures and burn-ups for nitride and carbide fuels, it is important to know which higher phases might form. Phases present in TRISO fuels during irradiation directly impact the migration and reactions of the fission products generated within the kernel, which in turn impact the silicon carbide (SiC) layer integrity through mechanical stress and chemical degradation^14^. Of course, real fuels consist of a composite of different structures and stoichiometries due to non-equilibrium fabrication regimes.

Higher nitrides in the U–N system are known, including uranium sesquinitride (U_2_N_3_)^3,15^ and uranium dinitride (UN_2_) which exists in the hypostoichiometric form UN_2−x_ with experimental studies defining the ratio of nitrogen-to-uranium atoms to be greater than 1.75^15^. Stoichiometric U_2_N_3_ undergoes a phase transition from cubic α ($Ia\overline{3}$) to trigonal β ($P\overline{3}m1$) at 1073 K^15^. Uranium carbide is also known to exhibit a sesquicarbide phase (U_2_C_3_) and similarly uranium dicarbide is only observed in hypostoichiometric form (UC_2−x_) with a carbon-to-uranium atomic ratio of 1.77–1.96^16^. These structures are believed to be highly nonstoichiometric due to fabrication and irradiation conditions. A key point of difference with the nitrides is that UC_2−x_ is only predicted to be stable at temperatures above ~ 1750 K^17,18^ and exists in two different structural forms: tetragonal α-UC_2−x_ (1753–2050 K) and cubic β-UC_2−x_ (> 2050 K)^19^.

Analogous sesquicarbide and low- and high-temperature dicarbide structures exist in the Pu-C system; however, plutonium monocarbide is only hypostoichiometric (PuC_1−x_)^17^ and while a triplutonium dicarbide (Pu_3_C_2_^20^) phase is reported, its crystal structure has not been identified experimentally^21^. In contrast to the other systems, plutonium nitride appears to exhibit only the mononitride phase with limited nonstoichiometry^22,23^.

For uranium nitride, density functional theory (DFT)-based simulations have been performed for over forty years. Brooks and Kelly^24,25^ applied the Full-Potential Linearised Muffin-Tin Orbital (FP-LMTO) method to characterise the structural and magnetic properties of uranium mononitride. These studies were subsequently followed by UN characterisation DFT simulations using linear combination of atomic orbitals (LCAO) and plane-wave (PW) DFT methods^26–28^ with covalent bonding better characterised by the former and metallic bonding by the latter. These methodologies were also applied to consider basic lattice properties of U_2_N_3_ and UN_2_^29–31^ with results in good agreement with available experimental data. This work has been followed by several studies further interrogating the structural and electronic properties of the U–N phase space. Weck et al*.*^32^ investigated the lattice parameters and bond lengths in UN and UN_2_ using a generalized gradient approximation (GGA) exchange correlation functional. However, the experimental data for UN_2_ referenced in this study does not identify any nonstoichiometry in the samples synthesised, and the study did not seek to confirm the structural stability of the stoichiometric form of the dinitride. Jin et al*.*^33^ validated the importance of including a Hubbard parameter (GGA + U) in the system Hamiltonian for U_2_N_3_ to improve the characterisation of 5f electrons when comparing their results with experiment. In addition, their exploration of magnetic configurations for this structure indicates its impact is negligible in predicting structural and electronic properties. Obodo and Braun^34^, also using GGA + U, confirm the influence of nitrogen vacancies on the stability of UN_2_ and obtain a nitrogen-to-uranium atomic ratio consistent with experimental observations. The phase transitions in the U–N system were modelled by Wang et al*.*^35^ using the non-DFT method of cluster formula theory to examine site occupations in the different crystal structures and identify the differences between structure bonds and angles which define the various phase transitions.

Theoretical studies of the Pu–N system are less extensive relative to U–N given the lack of structural data for higher plutonium and nitrogen phases. Yang et al*.*^36^ and Wen et al*.*^37^ reported structural, electronic and magnetic properties of PuN using hybrid functionals. These studies obtained the correct crystal structure and magnetic configuration^38^ and show the dominance of the Pu 5f electrons at the Fermi level. Lai et al.’s^39^ study investigated nonstoichiometry in PuN using GGA + U calculations with special quasirandom structures (SQS) in which they confirmed that the structure is only stable in its stoichiometric form. Obodo and Chetty^40^ and Wen et al.^37^ performed structural characterisation studies for a range of actinide mononitrides and dinitrides, including plutonium, where the former discovered a form of PuN_2_ (*P*4/mmm) with elastic and dynamic stability, contrary to existing experimental evidence.

The characteristics of uranium monocarbide have been studied extensively using DFT over the past decade. Wdowik et al*.*^41^ accurately captured the structural, electronic and dynamic properties of UC through comparison with experimental x-ray photoemission and inelastic neutron scattering experiments. This was achieved using a fine-tuned Coulomb repulsive interaction term in a GGA + U scheme and including relativistic effects through the application of a spin–orbit interaction term. Shi et al*.*^42^ also achieved good agreement with experimental data for UC using the GGA + U scheme alone, which they subsequently extended to the investigation of U_2_C_3_ and UC_2_^43^. They identified their respective body-centred tetragonal and body-centred cubic structures and confirmed the mixture of covalent and metallic bonding through analysis of density of states. No computational studies have explored the substoichiometric range observed experimentally in UC_2_^16^.

Investigations into the Pu-C system using DFT methods have largely focused on the effect that carbon vacancies have on the physical properties of substoichiometric PuC. Yang et al*.*^44^ provided a direct comparison between structural, electronic and optical properties in stoichiometric and substoichiometric PuC using hybrid functionals. Lai et al*.*^45^ accurately predicted the experimental range of carbon vacancies in large SQS PuC_1−x_ structures in the GGA + U scheme and hypothesised that the increased thermodynamic stability of the carbon-deficient structures is due to a rearrangement of bonding surrounding the vacant sites on the carbon sublattice. However, this study incorrectly assumed ferromagnetic order in PuC. Yang has also performed studies on Pu_2_C_3_^46^ and PuC_2_^47^ using GGA + U functionals to successfully validate experimental lattice parameters, elastic constants and bonding properties.

The purpose of the present study is to predict which phases of these nitrides and carbides will be apparent at a given metal-to-nitride/carbide ratio at low temperature (i.e. based on enthalpy values). This is achieved by using spin-polarised DFT calculations to predict the ground state energies of different phases with respect to the metal and nitrogen or graphite reference states. Thus, uranium or plutonium nitrides and carbides are considered across the compositional range from 0 to 1, where, if M = U or Pu and A = N or C and a composition is described by formula M_x_A_y_, the compositional value is given by $\frac{x}{x + y}$. The stable phase or phases that will be apparent are identified by plotting the energies of each phase as a function of compositional range to generate thermodynamic stability (or formation energy) convex hull diagrams^48^, one each for uranium nitride, plutonium nitride, uranium carbide and plutonium carbide systems.

## Methodology

### Computational methodology

DFT calculations were performed using the VASP (Vienna ab initio Simulation Package) package^49^ employing plane-wave basis sets and projected augmented wave (PAW) pseudopotentials^50^. All DFT calculations were performed with the spin-polarised generalised gradient approximation (GGA) in the form of the Perdew–Burke–Ernzerhof (PBE) functional^51^. A cut-off energy for the plane-wave basis set of 500 eV was selected for all calculations. Monkhorst–Pack^52^ k-point meshes with k-point densities of 0.04 Å^−1^ were applied to all the conventional cells with converged k-point meshes subsequently applied to structures within 10 meV of the convex hull. The energy and force convergence criteria for the relaxed structures were set at 10^–7^ eV and 10^–3^ eV/Å respectively. Electron occupancies of all compounds were initially described using Gaussian smearing with a smearing width of 0.05 eV. Given the metallic nature of these compounds, Methfessel–Paxton smearing^53^ was subsequently performed on all stable structures and any metastable structures within 10 meV of the convex hull. Smearing parameters for each of these structures were determined by convergence testing, with the optimal value chosen when the difference between the total electronic energy and the electronic free energy was less than 1 meV/atom.

It is known that local-density approximation (LDA) and GGA functionals incorrectly predict the ground states of UN and PuN to be ferromagnetic^54^, due to their inability to describe the strongly correlated 5f electrons of the actinide ions. The DFT + U method is used to address this without adding significant computational cost. This study applies values of U = 2.40 eV and J = 0.50 eV to the f-orbitals as in the Dudarev et al*.*^55^ formalism for UN and UC, as seen in Gryaznov et al*.*^56^ and Lu et al*.*^57^, and U = 3.20 eV and J = 0.00 eV for PuN and PuC. Similar U and J (or U_eff_ = U–J) parameters have been applied in several DFT + U of these actinide compounds^31,37,39,45,58–62^. U-ramping^63,64^ has been applied to demonstrate that the applied U_eff_ values provide the correct magnetic orderings for UN and PuN (see S1 in the electronic supplementary information (ESI)), verifying the choice of parameters. The approach is validated later by comparison of results with and without a U_eff_ parameter.

All structures were sourced from experimental studies^65–71^ and the OQMD^72,73^ and Materials Project^74^ databases. In addition, where structures were not present within these databases, symmetrical analogues were created based upon the lattice configuration of the respective actinide compound.

To generate nonstoichiometric plutonium monocarbide structures, a Monte-Carlo algorithm^75^ was used to create SQS structures of 64-atom supercells of PuC_1−x_ (with the supercell size based on a previous study^45^) with values of x ranging between 0 and 0.35 in increments of 1/32 (corresponding to the composition range of 0.4 to 0.5).

Bader charge analysis^76^ was used to examine the charge distributions of the atoms in the compounds to examine the nature of the bonding between the actinide ions and nitrogen or carbon atoms.

### Chemical potentials

In determining the chemical potential for nitrogen, complications arise in DFT (GGA) calculations of the nitrogen molecule (N_2_) and solid nitrogen (N(s)) states due to overbinding^77^ and inaccurate descriptions of van de Waals interactions respectively. Reference states which are suitable for accurate first-principles calculations are required, hence the chemical potential for nitrogen was calculated using the method of Finnis et al*.*^78^. This method uses the known formation energies of metal binary nitrides under standard temperature and pressure, $\Delta G_{f}^{{M_{\alpha } N_{\beta } }} \left( {P_{{N_{\beta } }}^{^\circ } ,T^{^\circ } } \right):$

1 $$\Delta G_{f}^{{M_{\alpha } N_{\beta } }} \left( {P_{{N_{\beta } }}^{^\circ } ,T^{^\circ } } \right) = \mu_{{M_{\alpha } N_{\beta } }}^{DFT} - \alpha \mu_{{M_{\left( s \right)} }}^{DFT} - \beta \mu_{N} \left( {P_{{N_{\beta } }}^{^\circ } ,T^{^\circ } } \right)$$

where $\mu_{{M_{\alpha } N_{\beta } }}^{DFT}$ is the DFT total energy of the binary metal nitride, $\alpha \mu_{{M_{\left( s \right)} }}^{DFT}$ is the DFT total energy of the metal cation, and $\beta \mu_{N} \left( {P_{{N_{\beta } }}^{^\circ } ,T^{^\circ } } \right)$ is the chemical potential (or reference energy) of nitrogen under standard temperature and pressure. Metals without open d- and f-electron shells were chosen for this methodology because the self-interaction error inherent within DFT is less prominent for these systems. Therefore, we calculated the reference energy of nitrogen as the average of the chemical potentials of nitrogen in binary nitrides with weaker electron correlations. All values are provided in S2 of the ESI.

The reference state for carbon was determined by calculating the energy of a single carbon atom in bulk diamond and applying a correction term (− 0.017 eV/C atom) equivalent to the experimental difference in ground state energies between bulk diamond and graphite^79^. This method was applied given the inability of DFT exchange–correlation functionals, such as GGA, to accurately describe van de Waals interactions between graphite layers^80^, whereas this is not the case for diamond^81^. This methodology is widely used in other studies^82–86^.

The reference states for uranium and plutonium were chosen to be α-U and α-Pu metal respectively.

### Metastable states

As mentioned previously, the DFT + U method is required to create an energy penalty for electron delocalisation given DFT’s tendency to favour delocalisation in strongly correlated systems. However, given the preference of integer over partial orbital occupation in DFT + U, a system may converge to a metastable state given the possible range of f-orbital occupation configurations. There is a risk that the system will become trapped in these local energy minima given a sufficiently high energy barrier caused by the energy penalty imposed by the methodology as the system explores partial occupancies between states. This could result in DFT + U not accurately identifying the true ground state of a system. Thus, here the occupation matrix control (OMC) methodology, developed by Dorado et al*.*^87^ and implemented as a package in VASP by Allen and Watson^88^, is applied to study the impact of electron occupation. This method trials all possible integer f-orbital configurations for a system, with each diagonal occupation matrix fixed for 15 electronic self-consistency steps in a structural relaxation simulation to guide the electrons towards the initially specified occupation configuration. Furthermore, structural relaxations are performed without the occupation constraint to identify the ground state configuration.

OMC is not universally applied across all structures given the significant computational cost. Given these constraints, OMC was only applied to known experimental structures which resided on or are within 10 meV of the thermodynamic stability curves.

### Convex hull of thermodynamic stability

A simple assessment of thermodynamic stability of compounds can be performed using DFT ground state energies and (global) convex hull analysis. All calculations are performed in the absence of temperature (including entropic) and pressure effects thus allowing the Gibbs free energy to be equated with the total DFT ground state energy. A material is defined as thermodynamically stable if, under a defined set of conditions, its energy cannot be lowered through a rearrangement of atoms via decomposition (separation of phases) or combination (creation of a polymorph). The formation energy of each compound $\left( {\Delta E_{f} } \right)$ is calculated using its DFT ground-state energy and the respective energies of its elemental phases. For example, in the case of uranium nitride compounds:

$$\Delta E_{f} = E_{{U_{\alpha } N_{\beta } }} - \alpha E_{U} - \beta E_{N}$$

where $E_{{U_{\alpha } N_{\beta } }}$ is the DFT ground-state energy of the compound, $E_{U}$ is the reference state (or chemical potential) of uranium, and $E_{N}$ is the reference state of nitrogen. Formation energies greater than zero indicate compounds which are unstable with respect to metal and nitrogen or metal and graphite (i.e. there is no thermodynamic driving force to create a product more thermodynamically favourable than its constituent elements). Conversely, a negative formation energy indicates that the compound is either stable or metastable, with the most stable compounds lying on a convex curve connecting the phases on the lowest-energy envelope, which is referred to as the convex hull of thermodynamic stability^48^.

## Results

This study focuses on the relative stability of phases across the compositional ranges through predictions of the convex hulls. Others have previously successfully predicted basic lattice properties for many of the phases investigated here. As such, these basic properties and lattice structure symmetries and parameters of those phases are reported in S5 of the ESI.

### Actinide nitrides

**Fig. 1:**
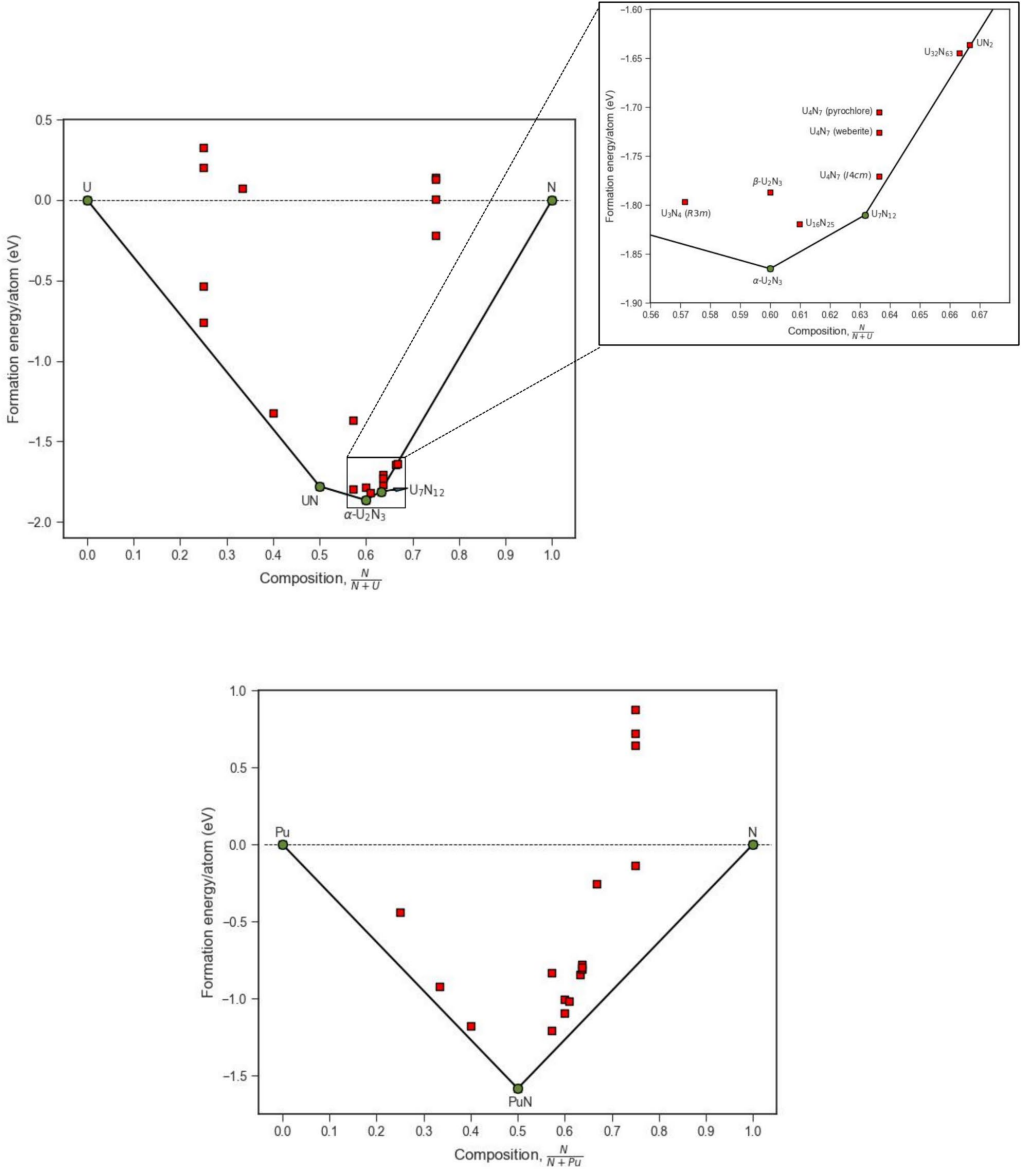
Thermodynamic convex hull for uranium (top) and plutonium (bottom) nitride compounds, with stable compounds on the lowest-energy envelope represented as green filled circles. Metastable structures which were also modelled are represented as red filled squares below the horizontal dotted line and above the line are not stable with respect to their constituent elements.

**Fig. 2:**
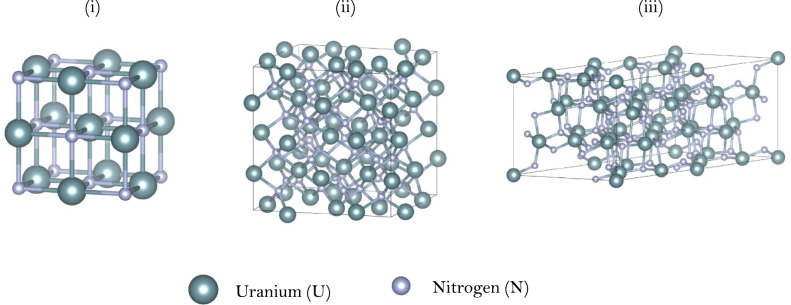
Predicted stable compounds of (i) uranium mononitride (UN) (ii) uranium sesquinitride (α-U_2_N_3_) and (iii) uranium nitride delta phase (U_7_N_12_); plutonium nitride (PuN) exhibits the same crystal structure as UN.

The global convex hulls for uranium and plutonium nitrides are shown in Fig. 1, with the stable ground state structures shown in Fig. 2. For the uranium–nitrogen system there is no predicted stable structure with a nitrogen content less than UN, which exhibits the rocksalt ($Fm\overline{3}m$) structure. The first higher nitride is α-U_2_N_3_ with a bixbyite cubic structure ($Ia\overline{3}$). The β-U_2_N_3_ phase is predicted stable but correctly identified at a higher energy, 78.04 meV/atom above the hull (reflecting the experimental α → β transformation temperature of 1073 K^89^). Beyond that, a substoichiometric UN_2−x_ structure is identified with composition U_7_N_12_ (see Fig. 2iii). While these predictions are in general agreement with experimental observations^3,15^, no experimental or theoretical studies have explored a UN_2−x_ configuration existing as an ordered derivative of the defect fluorite structure. While U_7_N_12_ is identified specifically on the hull, several other configurations and stoichiometries lie close to the convex hull and will be described in the discussion section below.

For the plutonium-nitrogen system, only PuN is predicted to lie on the convex hull (see Fig. 1), in agreement with experimental observations. That is, while many other structures were modelled, all lie above the line of thermodynamic stability.

### Actinide carbides

**Fig. 3:**
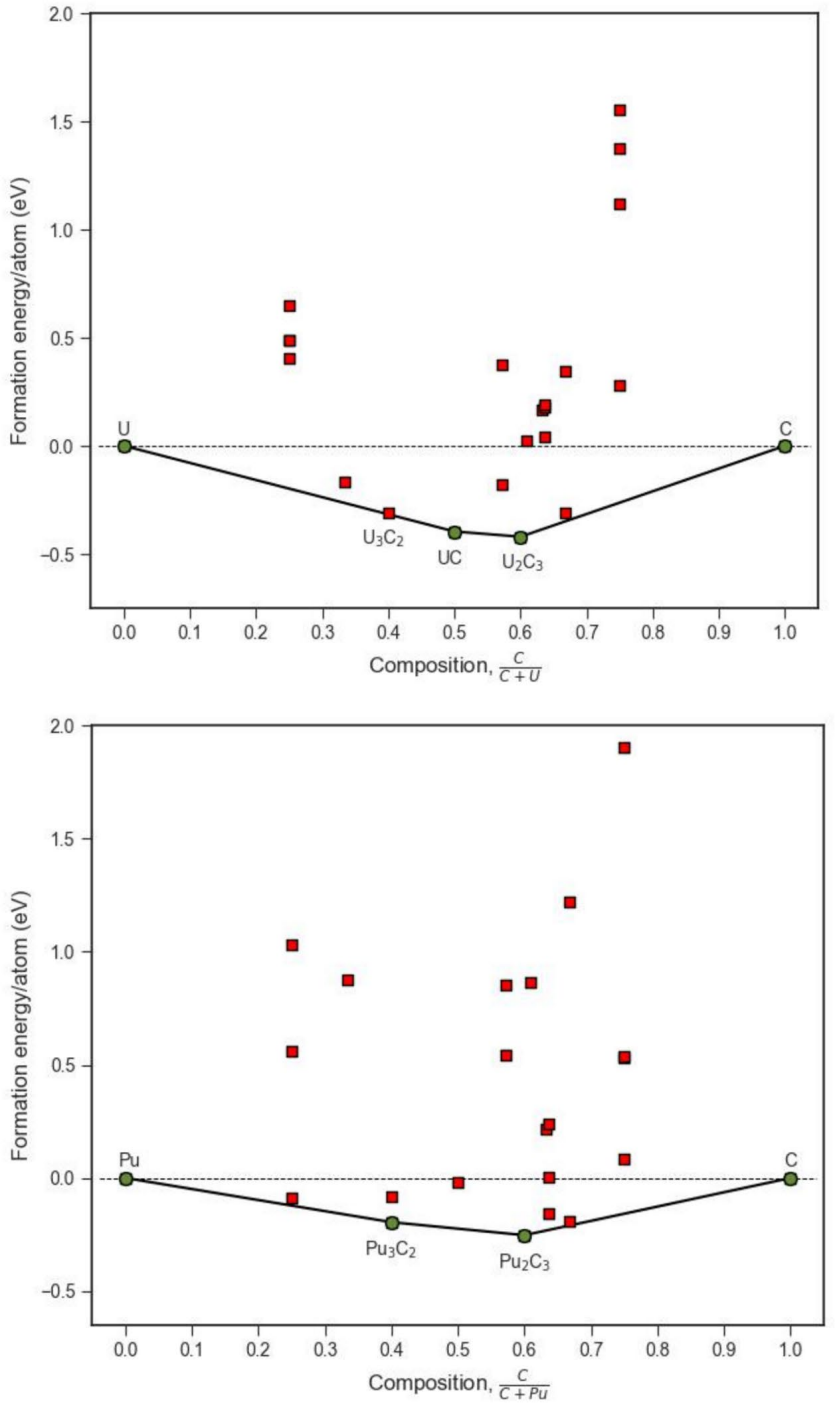
Thermodynamic convex hull for uranium (top) and plutonium (bottom) carbide compounds with stable compounds on the lowest-energy envelope represented as green filled circles. Metastable structures which were also modelled are represented as red filled squares below the horizontal dotted line and above the line are not stable with respect to their constituent elements.

**Fig. 4:**
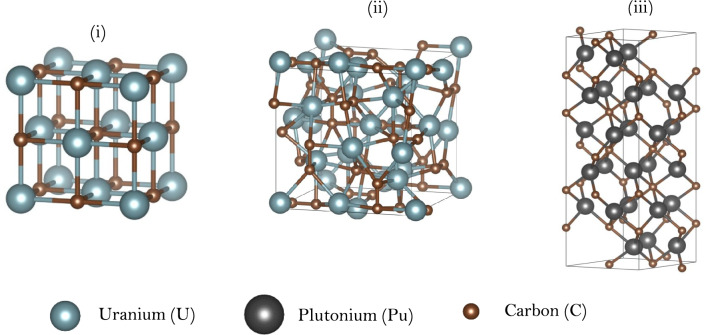
Predicted stable compounds of (i) uranium moncarbide (UC), (ii) uranium sesquicarbide (U_2_C_3_) and (iii) triplutonium dicarbide (Pu_3_C_2_). Plutonium sesquicarbide (Pu_2_C_3_) exhibits the same crystal structure as U_2_C_3_.

The convex hulls for uranium and plutonium carbides are shown in Fig. 3, with the stable ground state structures shown in Figs. 4. As predicted for uranium nitrides, both UC ($Fm\overline{3}m$) and U_2_C_3_ ($Ia\overline{3}$) are present on the convex hull and observed experimentally. Both exhibit the same structures observed in the U–N system. However, there is also a structure at lower carbon content (U_3_C_2_) which, while not quite predicted to sit on the convex hull, lies very close with a decomposition energy of only 1.93 meV/atom. Conversely, although substoichiometric UC_2−x_ (fluorite) structures have been identified experimentally, they are only observed above 1750 K^17,18^. This is consistent with results shown in Fig. 3, where fluorite-based substoichiometric structures lie significantly above the convex hull in the U_2_C_3_–UC_2_ phase region (but were close to the convex hull in the U–N diagram—see Fig. 1). Thus, the convex hull for uranium carbides is distinct from that for uranium nitrides.

The convex hull for plutonium carbides (Fig. 3) is also quite different to the simple hull for plutonium nitride. Here, two plutonium carbides are predicted to sit on the convex hull. Firstly, Pu_3_C_2_ with the same structure to that predicted for U_3_C_2_ (which was close to but not on its convex hull) and secondly, Pu_2_C_3_ which again is structurally identical to U_2_C_3_. An important difference between plutonium systems is the absence of a stoichiometric monocarbide on the convex hull. However, experimental studies report that PuC is nonstoichiometric (PuC_1−x_) with a range of x values between 0.08 and 0.25^90,91^. This will be explored in the discussion section below.

## Discussion

### Inclusion of a Hubbard parameter and comparison with experimental observations

Based on enthalpies calculated using the DFT + U approach, Figs. 1 and 3 illustrate which structures are predicted to be observed, assuming entropy terms are not sufficient to change structure preferences. In all four systems the structures predicted are those observed experimentally. In the ESI, convex hulls are shown derived using only the DFT approach (i.e. all parameters are the same except for the omission of U_eff_ parameters). In these cases, the agreement with experimental observations is substantially worse (see S3 in the ESI). In particular, without a U_eff_ parameter, for uranium nitrides the α-U_2_N_3_ phase is not predicted as stable but conversely for the plutonium nitrides, this bixbyite sesquinitride structure is predicted to be stable. This comparison therefore largely validates the consistent application of a Hubbard parameter (DFT + U) to improve the characterisation of f-electron localisation in uranium and plutonium.

In addition to DFT + U, as commented by other others such as Claisse et al.^58^, the use of the occupation matrix control (OMC) scheme results in lower energies for all the structures where it was applied (relative to calculations where the effects of metastability are neglected). This is notably observed in the Pu–N system (Figs. 1 and S3 in the ESI) where this methodology confirms the absence of higher Pu and N phases, in agreement with experiment. This is contrary to other studies^37,40^ where a dinitride phase is predicted to exist based upon elastic and phonon properties. However, in those studies, DFT + U was used without a mechanism to reduce the likelihood of convergence towards metastable states. Furthermore, predictions were based on structure stability alone, that is, in the absence of relative formation energy analysis (e.g. convex hulls).

### Comparison of convex hulls

By comparing Figs. 1 and 3, it is apparent that the carbide convex hulls are shallower relative to the nitrides. This is a consequence of the lower thermodynamic driving forces in the formation of the actinide carbides compared with nitrides, with respect to the reference states. Considering bond formation, the Pauling electronegativity of a nitrogen atom is 3.04 compared to 2.55 for carbon^92^. This is reflected in the Bader charges predicted for nitrides which span values from − 1.42 to − 1.75 whereas for the carbides, values are − 0.95 to − 1.75.

Considering the electronegativities once more, the difference in electronegativity between the actinide nitrides is 1.66 and 1.76 for U–N and Pu–N, and for the carbides is 1.17 and 1.27 for U–C and Pu–C respectively. Thus, as the greater electronegativity difference suggests, the larger Bader charges indicate that stronger ionic bonding interactions are present in the nitride structures. Bader charges are a Quantum Theory of Atoms in Molecules (QTAIM) approach which measure the amount of charge transferred between the two atomic basins and hence are often used as an ionic bonding metric, but they have also been very strongly correlated to bond energies in other actinide complexes^93^. Further support of stronger bonding between the actinides and nitrogen is shown with the increased relative quantity of 5/6d and 2p hybridised states in the projected density of states plots in the ESI (Figures S4 and S5). 6d orbitals have been shown to engage in more covalent bonding in the early actinide series than their 5f counterparts, due to their more spatially diffuse behaviour^94,95^. Thus, this indicates that the increased stability witnessed for the nitrides may be achieved through the formation of covalent interactions also. However, note that the change in stability of phases discussed above, with and without the U_eff_ parameter applied to the f-orbitals, highlights that the influence on the f-orbitals in bonding should not be neglected.

### Contribution to structure identification

Many of the crystal structures on the convex hulls are already identified experimentally and the current study consistently reproduces this data (see S5 in the ESI for details). There are, however, three where the current simulations offer new insight: UN_2−x_, Pu_3_C_2_ and related to this, PuC_1−x_. These will now be considered in turn.

**Fig. 5:**
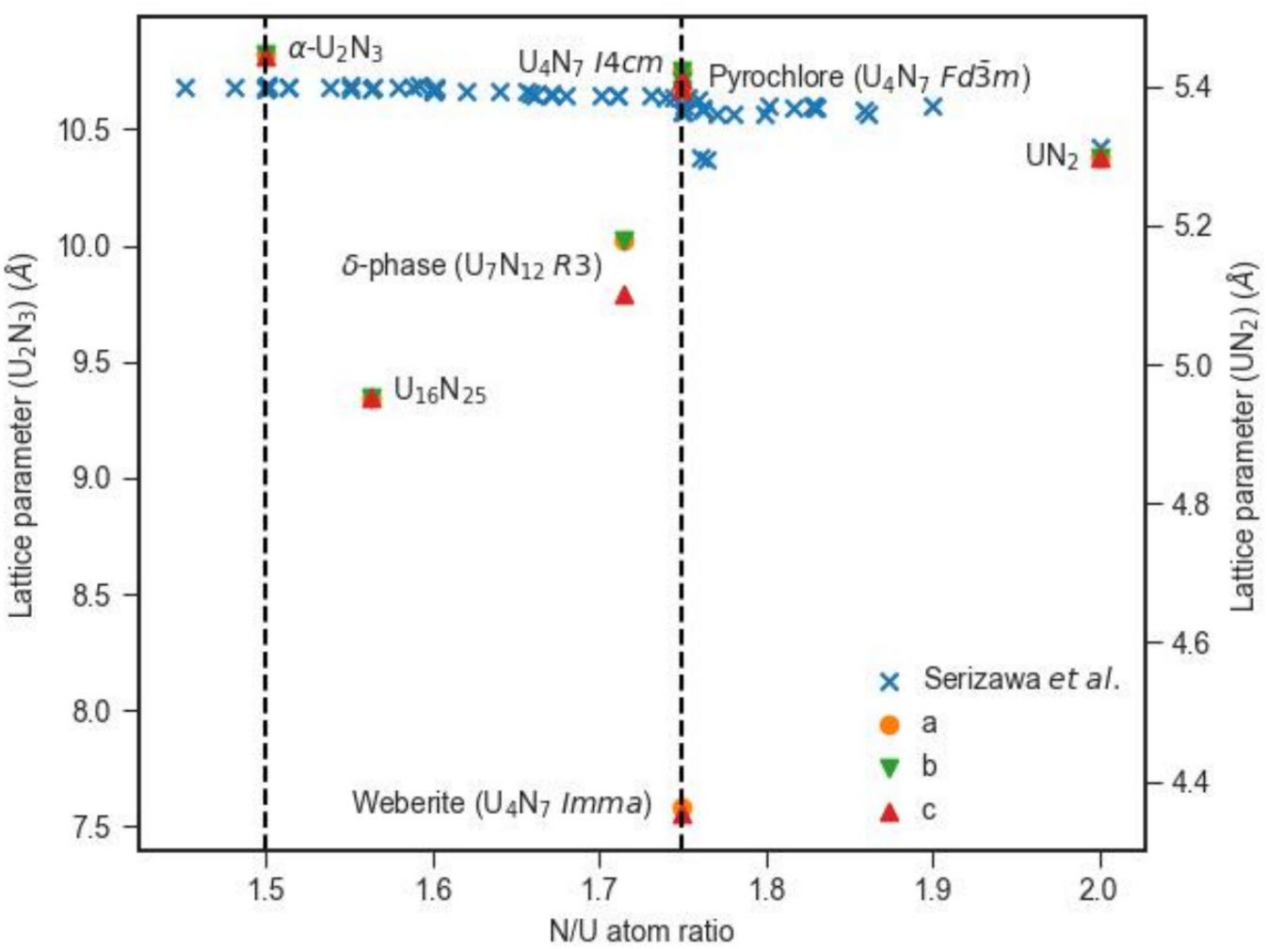
Variation of predicted unit cell lattice parameters (a, b, and c) for structures within the α-U_2_N_3+x_ to UN_2−x_ composition range. Comparison is made to the experimental data reported by Serizawa et al*.*^96^. The black vertical dotted lines represent the stoichiometric compositions of α-U_2_N_3_ (N/U = 1.5) and U_4_N_7_ (N/U = 1.75).

Starting with UN_2−x_, previous simulation studies^30,32,34^ assumed a stoichiometric composition UN_2_ with a fluorite structure ($Fm\overline{3}m$). However, Rundle et al*.*^65^ stated in 1948 that the maximum nitrogen-to-uranium ratio is around 1.75. Furthermore, Rundle et al*.* suggested previous reporting of compounds at this composition range (such as U_4_N_7_) provided further confidence in their reported non-stoichiometric range in UN_2−x_. It was established that the lattice parameter of UN_2−x_ decreased as a function of increasing nitrogen content (i.e. decreasing value of x in UN_2−x_), though the magnitude of the decrease was marginal, from 10.68 Å at N/U = 1.54 to 10.63 Å at N/U = 1.75 (see Fig. 5).

Here calculations are performed on a range of compositions in the phase region between U_2_N_3+x_ and UN_2−x_. A family of crystal structures related to defect fluorite derivatives in metal oxides are simulated, including pyrochlore (U_4_N_7_ $Fd\overline{3}m$), weberite (U_4_N_7_ *Imma*) and the delta phase (U_7_N_12_ *R3*). Figure 5 displays the lattice parameters of simulated and experimental structures as a function of the nitrogen-to-uranium atomic ratio within the U_2_N_3+x_-UN_2−x_ range.

The experimental data presented in Fig. 5 is a combination of U_2_N_3+x_ and UN_2−x_ lattice parameter data^96,97^ with vertical dotted lines drawn for the stoichiometric compositions of the known experimental phase of α-U_2_N_3_ and the reported phase of U_4_N_7_^65^. Despite the similarity in values, not all experimental studies identify a phase transition from Mn_2_O_3_-type to fluorite-type lattices at the U_4_N_7_ composition line—Serizawa et al*.*^96^ obtained X-ray diffraction patterns with peaks characteristic of the bixbyite structure at compositions of 1.75 and 1.80. However, this is contrary to several other studies^15,97^ where it was proposed that the change in gradient at N/U = 1.75 signifies a phase change to the defect fluorite lattice.

Despite the convex hull indicating the δ-phase structure was the most stable atomic configuration within the U_2_N_3+x_-UN_2−x_ region of U-N phase space, it has a trigonal crystal structure which is contrary to the cubic symmetry observed and predicted for α-U_2_N_3_ and UN_2_ respectively. In addition, its unit cell lattice parameters are several tenths smaller than the experimental values measured at its composition. However, the predicted lattice parameter for the pyrochlore and U_4_N_7_ *I4cm* structures (see Fig. 5) are close to experimental data, as is the value for stoichiometric UN_2_. In fact, UN_2_ sits very close to the convex hull, only 1.85 meV/atom above the line. Given the approximations of the computational method, an experimental observation of UN_2_ is not inconsistent with these calculations. Furthermore, at the necessary nitrogen partial pressure, if UN_2_ were fabricated, there would be negligible energy change driving the decomposition of UN_2_ as the material was quenched. This may account for its hypothetical identification upon review of experimental data^15,65,97,98^. Certainly, UN_2_ is a candidate for further consideration. While U_4_N_7_ *I4cm* is also close to the convex hull, it is not as close with a decomposition energy of 16.22 meV/atom. U_16_N_25_ is a little higher again (28.50 meV/atom) but also, the lattice parameter is rather low, and the symmetry is non-cubic. Finally, since U_4_N_7_ weberite and U_4_N_7_ pyrochlore have the same composition as U_4_N_7_ *I4cm,* they can be excluded given they have decomposition energies of 60.89 and 81.60 meV/atom respectively.

Considering this data as a whole, α-U_2_N_3_, U_4_N_7_ pyrochlore and UN_2_ and are all fluorite related, all sit close to or on the convex hull and span the stoichiometry range from the bottom of the convex hull at 0.60–0.67. Furthermore, the formation energy of U_32_N_63_ (a 2 × 2 × 2 UN_2_ fluorite supercell with one nitrogen vacancy) also sits very close to the convex hull. Thus, it is possible that a continuum of defective fluorite structures will form across this stoichiometry range with the exact composition reflecting the nitrogen chemical potential during fabrication (i.e. partial pressure of nitrogen at the fabrication temperature but also during the quench). With that in mind, it is important to reflect that here we use zero-temperature simulations in identifying structures within this composition space. More progress could be made by including temperature in the simulations by calculating vibrational and configurational entropy contributions, though it is beyond the scope of this study.

Turning to Pu_3_C_2_, a phase with this composition is reported^17^ and Fig. 3 shows Pu_3_C_2_ on the Pu-C convex hull. The predicted structure exhibits trigonal space group $R\overline{3}c$ No. 167 with Pu atoms at 18*e* and C atoms at 12c sites (hexagonal axes). The associated parameters for this structure are reported in Table 1. This structure was derived from a binary prototype structure for U_3_N_2_ in the OQMD database^72^ which defines prototype compounds at compositions where stability is predicted when comparing to similar binary compounds. An alternative crystal structure with $R\overline{3}m$ symmetry identified through a machine-learning model^99^ was also tested but did not sit on the convex hull. Interestingly, U_3_N_2_ (with these structures) was found to be unstable with respect to the U-N convex hull.

**Table 1:** Parameters for the predicted $R\overline{3}c$ Pu_3_C_2_ structure.

Space group	a (Å)	c (Å)	Atom	Wyckoff position	Coordinates
$R\overline{3}c$	6.43	18.03	Pu	18e	(0.6789, 0, 0.25)
			C	12c	(0, 0, 0.1549)

**Fig. 6:**
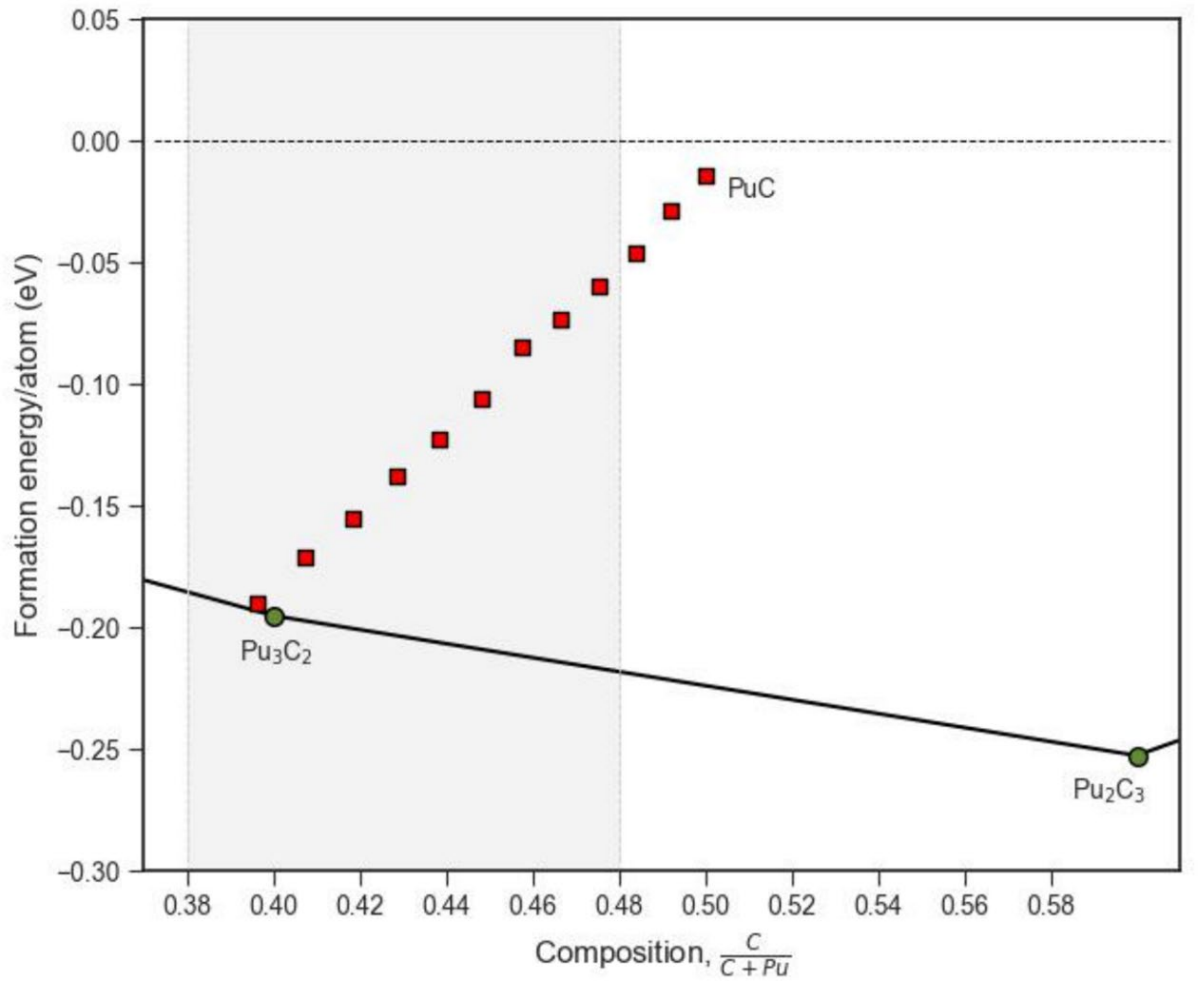
Convex hull of the Pu–C system in the region of Pu_3_C_2_ to Pu_2_C_3_. Red filled squares indicate the nonstoichiometric PuC_1−x_ SQS structures. Grey shaded region indicates the experimental hypostoichiometric range for PuC_1−x_^101^.

Lastly, we return to the confirmation (in Fig. 2) that stoichiometric PuC (rocksalt) is unstable at low temperatures as reported in existing experimental^90,91^ and computational studies, but that predicted phase diagrams^20,21,100^ show a broad carbon deficient nonstoichiometric region extending between PuC_0.6_ to PuC_0.92_. Figure 6 displays the convex hull for the Pu–C system in the phase region between Pu_3_C_2_ and Pu_2_C_3_, which encompasses all simulated nonstoichiometric PuC_1−x_ structures.

Figure 6 shows that the predicted formation energy of stoichiometric PuC is well above the convex hull. However, the formation energies of the nonstoichiometric PuC_1−x_ structures decrease linearly (overall) as a function of carbon vacancy concentration down to the experimentally identified composition corresponding to Pu_3_C_2_. The similarity of PuC_1−x_ and Pu_3_C_2_ formation energies may explain the difficulties in identifying the crystal structure of Pu_3_C_2_ and thus why it has been suggested its structure is related to the rocksalt structure, based upon X-ray diffraction patterns^90^.

## Conclusion

Results from a comprehensive study of U–N, Pu–N, U–C and Pu–C phase space have been presented. Formation energies of structures were calculated using the DFT + U methodology and stable phases identified with respect to end member compositions (i.e. U or Pu metal and nitrogen gas or graphite). Predictions of phases expected across the composition space were identified using convex hull analysis. Figures 1 and 3 show the four convex hulls along with the relative energies of other phases that were successfully simulated but of higher energy. Those higher energy phases should not be observed at low temperature in equilibrium phase diagrams but might become favourable at higher temperatures. The study highlighted the importance of incorporating an appropriately characterised U_eff_ to obtain predictions consistent with experimental findings. Without a suitable U_eff_ parameter some phases such as α-U_2_N_3_ did not sit on the convex hull while others such as α-UC_2_ did (compare data in Figs. 1 and 3 with Figure S2 and S3 in the ESI).

Overall, the convex hulls of the four systems are quite different, that is, different sets of compounds are stable. For example, for Pu–N only PuN (cubic rocksalt structure) sits between the Pu metal and nitrogen end members. Conversely, in the Pu–C system, Pu_3_C_2_ and Pu_2_C_3_ are predicted to sit on the convex hull but not stoichiometric PuC. However, the formation energy of Pu_3_C_2_ competes with a substoichiometric form of PuC (PuC_0.66_) which lies within the known stable range of nonstoichiometry of this monocarbide at low temperatures.

For both U–N and U–C systems, the rocksalt mononitride and carbide are the first structures predicted on the convex hulls, starting from the metal end member (although there is also a structure at lower carbon content (U_3_C_2_) which, while not quite predicted to sit on the convex hull, lies very close with a decomposition energy of only 1.93 meV/atom and is therefore worth further investigation, given energy contributions of higher temperatures and pressures). The next structures are α-U_2_N_3_ and U_2_C_3_ which are crystallographically identical. However, unlike Pu–N, U–N then transitions into a hypostoichiometric uranium dinitride (UN_2−x_) phase region. This was explored to ascertain whether any ordered defect fluorite crystal symmetries were feasible within the α-U_2_N_3+x_-UN_2−x_ continuous phase region. A δ-phase defect fluorite structure (U_7_N_12_) is predicted to lie on the convex hull, although its lattice dimensions are inconsistent with experimental data. Conversely, there is greater lattice structure agreement with the U_4_N_7_ structure (*I4cm*) but this lies 16.22 meV/atom above the hull. Stoichiometric UN_2_ lies only 1.85 meV/atom above the hull and is structurally comparable to experimental data, as is U_32_N_63_ which is 10.42 meV/atom above the hull. These predictions suggests that it is plausible for the stoichiometric form of UN_2_ and various substoichiometric compositions to be fabricated under the right conditions given such small decomposition energies are predicted and the uncertainties associated with the DFT + U methodology.

## Supplementary Information

Supplementary Information 1.

Supplementary Information 2.

## Data Availability

The datasets used and/or analysed during the current study are available from the corresponding author on reasonable request.
