# Supplementary material for: Structure stability of (U, Pu) C and (U, Pu) N compositions
Source: Sci Rep. 2025 Jun 6;15:19873. doi: 10.1038/s41598-025-03910-y (PMC12144085; doi:10.1038/s41598-025-03910-y)
Supplement: Supplementary file 1 — Supplementary Information 1. [file 41598_2025_3910_MOESM1_ESM.pdf]

Electronic Supplementary Information (ESI)

S1. Difference in ground-state energies for ferromagnetic and antiferromagnetic configurations

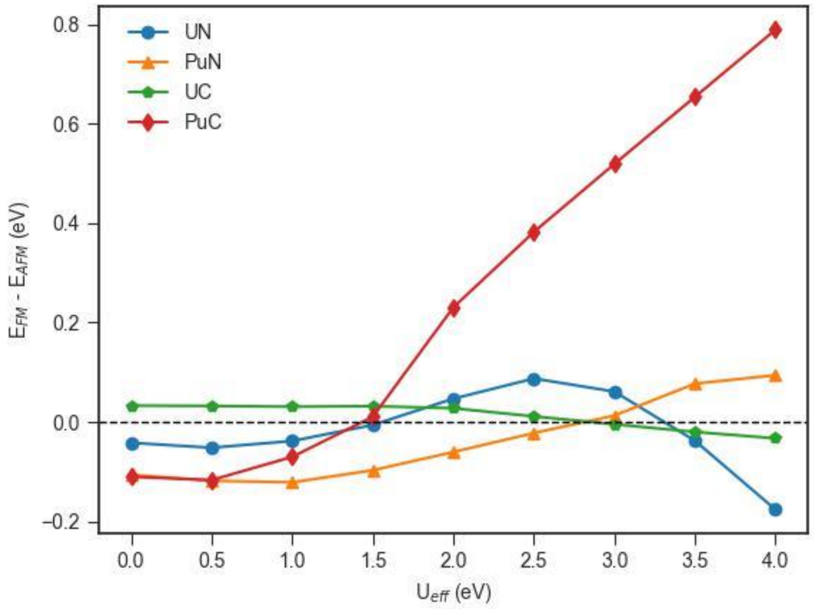

FIG. S1. Difference in ferromagnetic and antiferromagnetic ground-state energies as a function of effective Hubbard parameter (U_{eff}) for UN, PuN, UC, and PuC.

x-intercept values:

- UN: 1.56 and 3.31eV
- PuN: 2.82 eV
- UC: 2.86 eV
- PuC: 1.44 eV

S2. Chemical potential calculations

TABLE S1. List of structures and relevant DFT energies to calculate chemical potential for nitrogen (μ_N) using Equation 1.

**Structure**	**Experimental formation energy, ΔG_f (eV)**	**Compound DFT energy, $\mu^{DFT}_{M_\alpha N_\beta}$ (eV)**	**Cation DFT energy, $\mu^{DFT}_{M(s)}$ (eV)**	**Reference energy, μ_N(eV)**
ZrN	-3.79	-20.37	-8.52	-8.06
Ca_3N_2	-4.48	-26.42	-1.93	-8.07
Mg_3N_2	-4.79	-25.03	-1.51	-7.86
TiN	-3.50	-19.63	-7.84	-8.29
**Average μ_N (eV)**				-8.07

S3. Convex hulls for DFT-GGA simulations (i.e. without U_{eff} parameters)

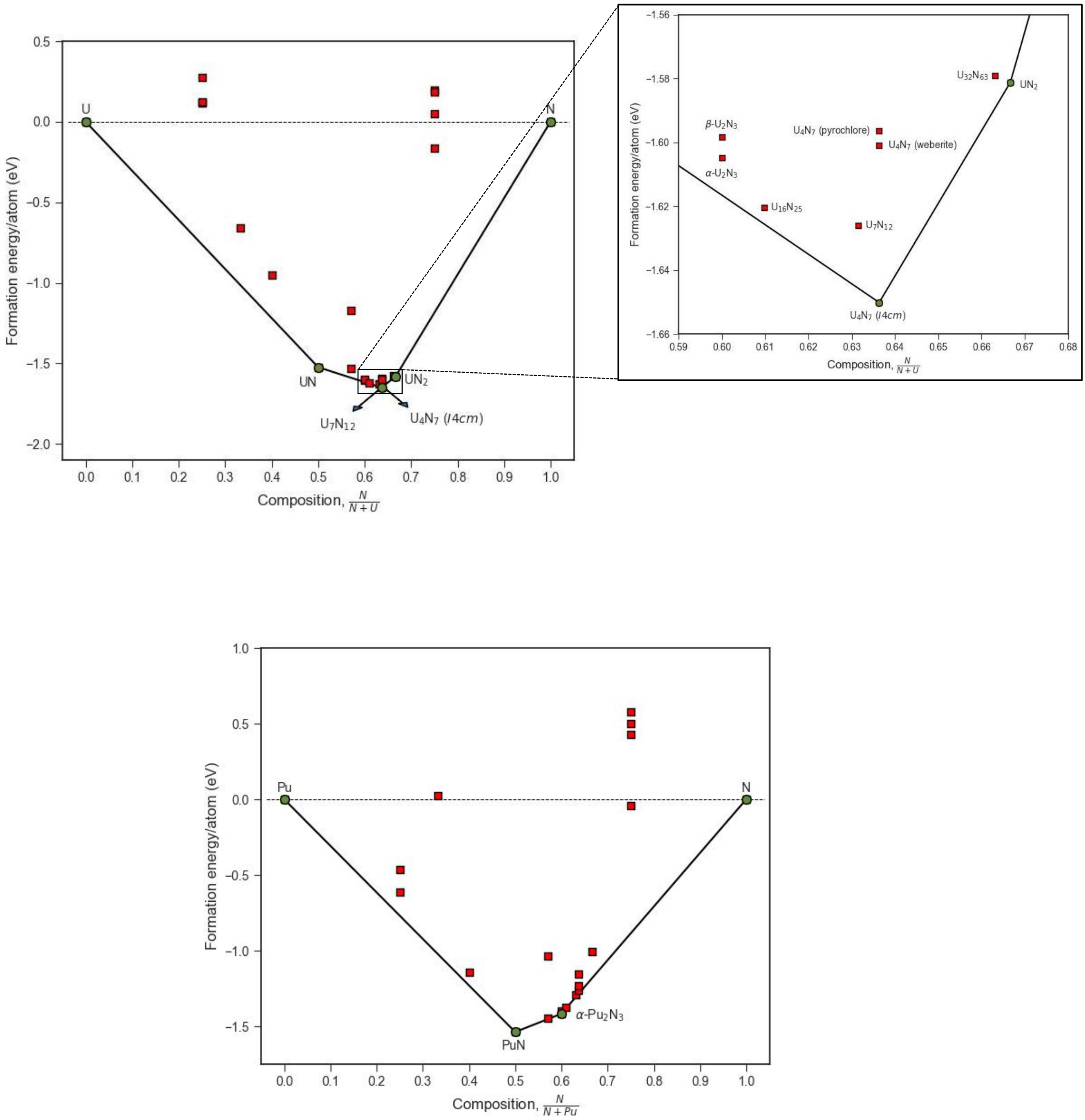

FIG. S2. Thermodynamic convex hull for uranium (top) and plutonium (bottom) nitride compounds with stable compounds represented as green filled circles. Unstable structures which were also modelled are represented as red filled squares.

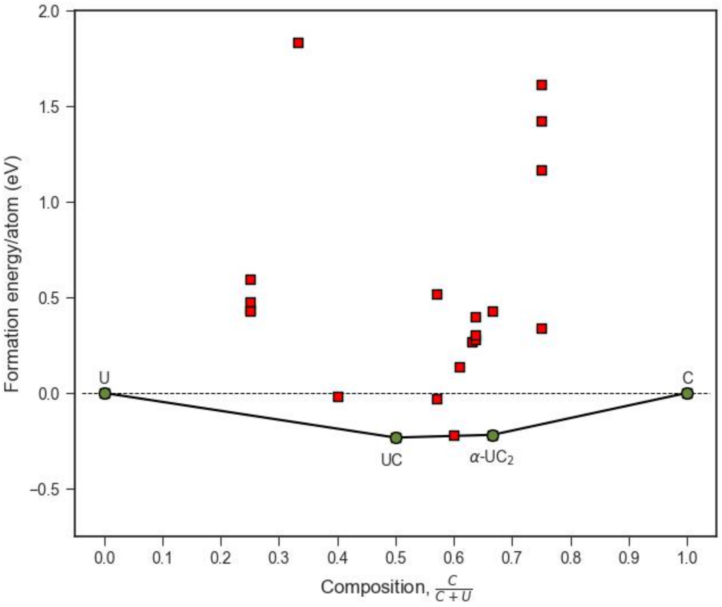

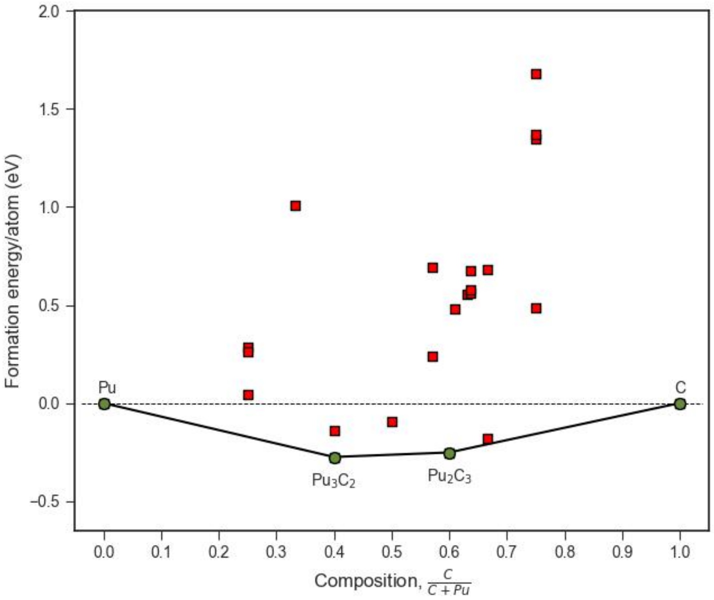

FIG. S3. Thermodynamic convex hull for uranium (top) and plutonium (bottom) carbide compounds with stable compounds represented as green filled circles. Unstable structures which were also modelled are represented as red filled squares.

S4. Projected density of states for all predicted stable compounds using GGA+U

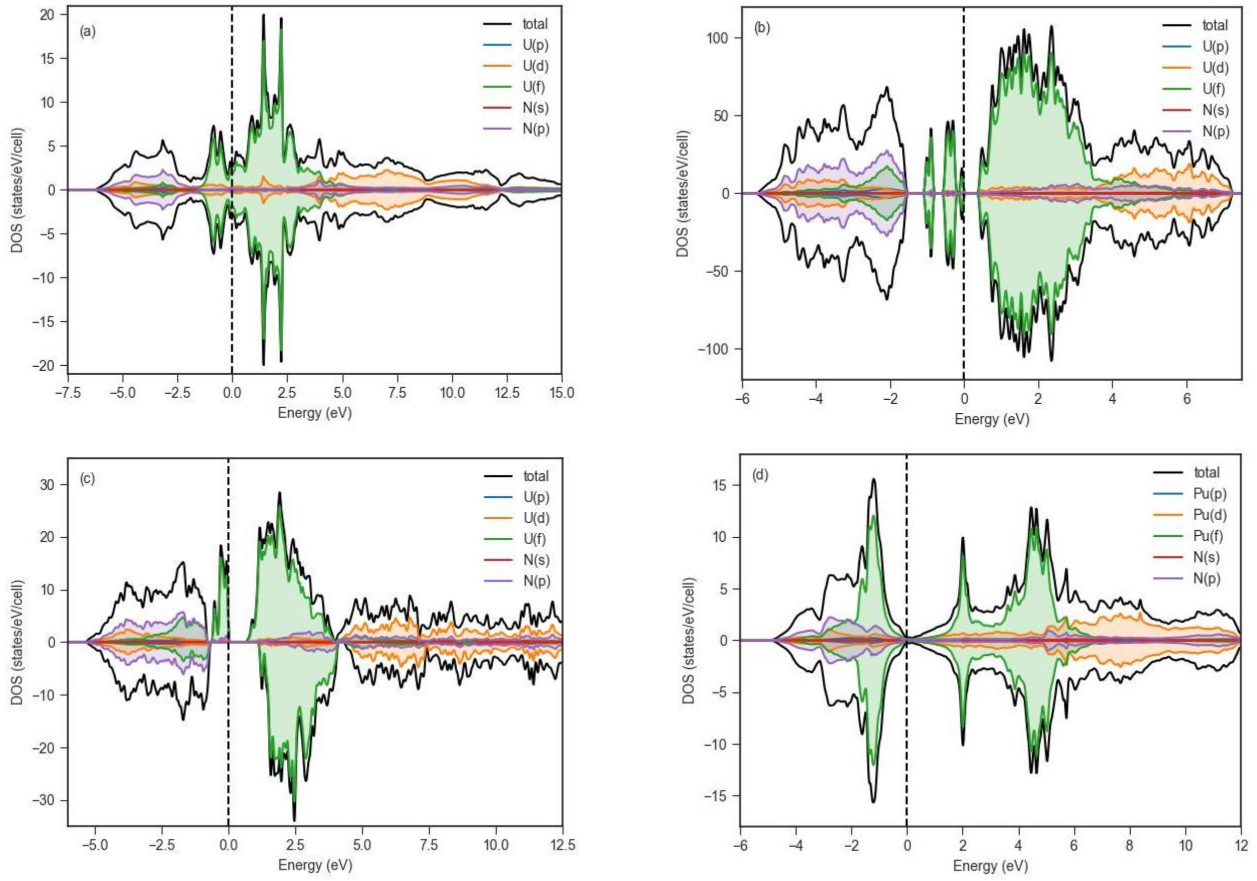

FIG. S4. – Total and projected density of states (DOS) for (a) UN, (b) α-U_2N_3, (c) U_7N_{12}, and (d) PuN.

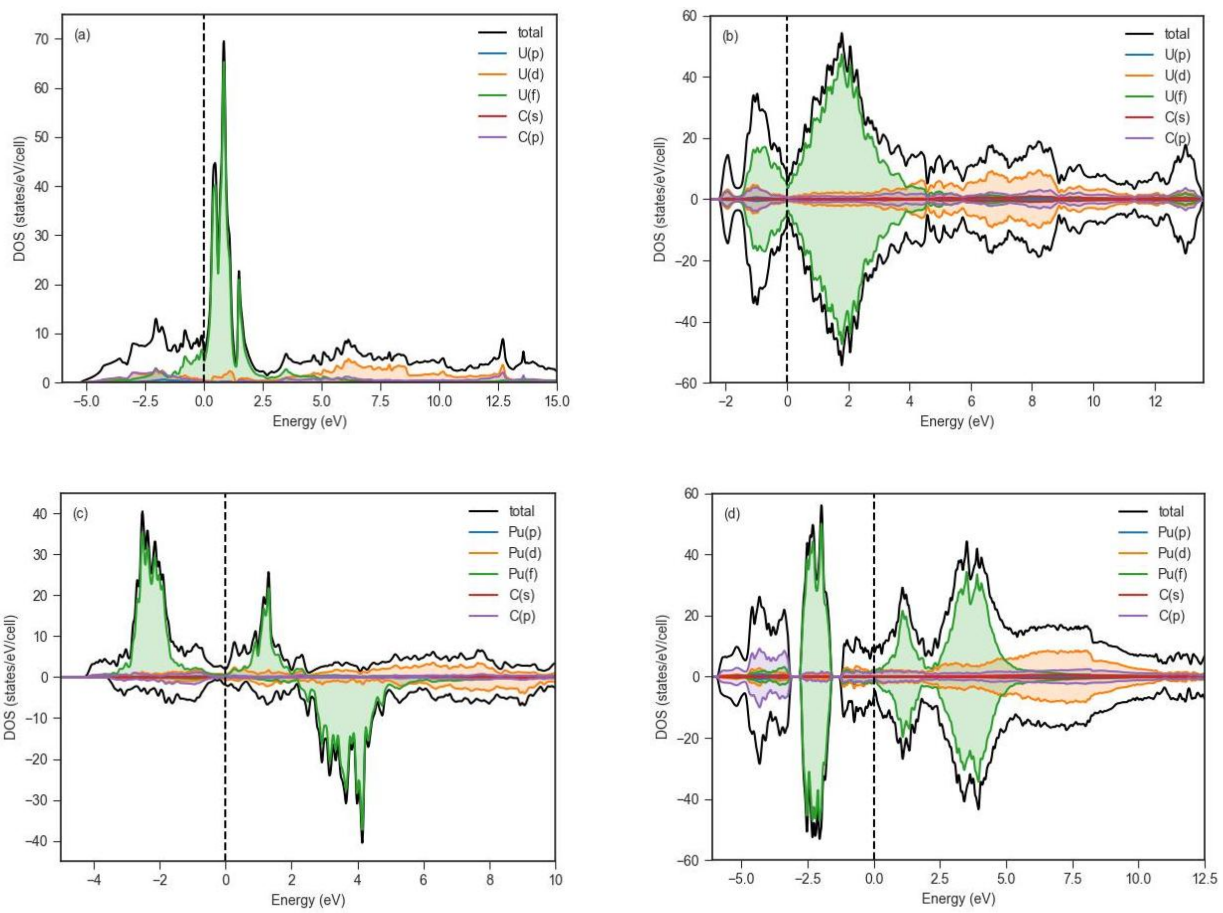

FIG. S5. Total and projected density of states (DOS) for (a) UC, (b) U_2C_3, (c) Pu_3C_2, and (d) Pu_2C_3.

S5. Structural information for predicted stable structures

TABLE S2. Structural information for stable compounds identified through DFT+U calculations. Experimental data, where known, is reported in a second row below the predicted values.

Structure	a	b	c	Magnetic state*	μ_B	$Q_{U/Pu}$	$Q_{C/N}$
UN	4.92	5.01	4.94	AFM	1.82, -1.82	1.75	-1.75
	4.89 [1]	4.89	4.89	AFM [2]	0.75 [2]		
U_2N_3	10.82	10.82	10.82	AFM	1.48, -1.48	2.28	-1.52
	10.68 [1]	10.68	10.68	AFM [3]			
U_7N_{12}	10.02	10.02	9.38	FM	1.09	2.43	-1.42
PuN	5.12	5.05	5.08	AFM	4.95, -4.95	1.64	-1.64
	4.91 [4]	4.91	4.91	AFM [5]			
UC	4.96	4.96	4.96	NM	0	1.71	-1.71
	4.96 [6]	4.96	4.96	PM [7]			
U_2C_3	8.19	8.21	8.18	AFM	1.78, -1.78	1.69	-1.12
	8.09 [3]	8.09	8.09	AFM [8]			
Pu_3C_2	6.43	6.42	18.03	FM	5.13	1.17	-1.76
Pu_2C_3	8.51	8.44	8.50	AFM	5.02, -5.12	1.44	-0.96
	8.13 [9]	8.13	8.13	PM [10]			

*FM = ferromagnetic, AFM = antiferromagnetic, PM = paramagnetic

[1] Rundle, R. E., Baenziger, N. C., Wilson, A. S. & McDonald, R. A. The Structures of the Carbides, Nitrides and Oxides of Uranium. *J. Am. Chem. Soc.* **70**, 99-105 (1948).
[2] Curry, N. A. An investigation of the magnetic structure of uranium nitride by neutron diffraction. *Proc. Phys. Soc.* **86**, 1193 (1965).
[3] Troc, R. Magnetic susceptibility of the uranium nitrides. *J. Solid State Chem.* **13**, 14-23 (1975).
[4] Muromura, T. Effect of Oxygen and Carbon Impurities on Lattice Parameter of PuN. *J. Nucl. Sci. Technol.* **19** (10), 852-854 (1982).
[5] Raphael, G. & de Novion, C. H. Susceptibilities magnetiques des mononitrures et sesquicarbures de thorium, uranium et plutonium. *Sol. State Comm.* **7** (10), 791-793 (1969).
[6] Austin, A. E. Carbon Positions in Uranium Carbides. *Acta Crystallogr.* **12** (2), 159-161 (1959).
[7] Brodsky, M. B. Magnetic properties of the actinide elements and their metallic compounds. *Rep. Prog. Phys.* **41** (10), 1547 (1978).
[8] Matsui, H. Magnetic Behavior of Neutron Irradiated U_2C_3. *J. Nucl. Sci. Technol. (Tokyo, Jpn.)* **18** (11), 895-897 (1981).
[9] Zachariasen, W. H. Crystal chemical studies of the 5f-series of elements. XV. The crystal structure of plutonium sesquicarbide. *Acta Crystallogr.* **5**, 17-19 (1952).
[10] Green, J. L., Arnold, G. P., Leary, J. A. & Nereson, N. G. Crystallographic and magnetic ordering studies of plutonium carbides using neutron diffraction. *J. Nucl. Mater.* **34**, 281-289 (1970).
